# Characterization of Hydroxyproline-Containing Hairpin-Like Antimicrobial Peptide EcAMP1-Hyp from Barnyard Grass (*Echinochloa crusgalli* L.) Seeds: Structural Identification and Comparative Analysis of Antifungal Activity

**DOI:** 10.3390/ijms19113449

**Published:** 2018-11-02

**Authors:** Eugene Rogozhin, Artur Zalevsky, Alexander Mikov, Alexey Smirnov, Tsezi Egorov

**Affiliations:** 1 Department of Molecular Neurobiology, Shemyakin and Ovchinnikov Institute of Bioorganic Chemistry of the Russian Academy of Sciences, Moscow 117997, Russia; aozalevsky@fbb.msu.ru (A.Z.); mikov.alexander@gmail.com (A.M.); ego@ibch.ru (T.E.); 2 Department of Chemical Studies with Biologically Active Compounds of Microbial Origin, Gause Institute of New Antibiotics, Moscow 119021, Russia; 3 Faculty of Bioengineering and Bioinformatics, Lomonosov Moscow State University, Moscow 119991, Russia; 4 Institute of Molecular Medicine, Sechenov First Moscow State Medical University, Moscow 119146, Russia; 5 Department of Plant Protection, Timiryazev Russian State Agrarian University, Moscow 127550, Russia; smirnov@timacad.ru

**Keywords:** hairpin-like peptides, plant immunity, *Echinochloa crusgalli*, proline/hydroxyproline substitution, fungistatic activity, *Fusarium solani*, 3D modeling, in vitro binding assays

## Abstract

Herein, we describe a modified form of the antimicrobial hairpin-like peptide EcAMP1, isolated from barnyard grass (*E. crusgalli*) seeds, which is structurally characterized by a combination of high-pressure liquid chromatography, mass spectrometry, and automated Edman sequencing. This derivate has a single amino acid substitution (Pro19Hyp) in the second α-helical region of the molecule, which is critical for the formation of the hydrophobic core and the secondary structure elements. Comparing the antifungal activity of these two peptides, we found that the modified EcAMP1-Hyp had a significantly weaker activity towards the most-sensitive plant pathogenic fungus *Fusarium solani*. Molecular dynamics simulations and in vitro binding to the commercial polysaccharides allowed us to conclude that the Pro-19 residue is important for binding to carbohydrates located in the spore cell wall and it chiefly exhibits a fungistatic action representing the hyphal growth inhibition. These data are novel and significant for understanding a role of α-hairpinins in plant immunity.

## 1. Introduction

Plant defense peptides have roles in innate immunity to biotic and abiotic environmental stress factors. Part of them is combined into families of PR-proteins that are constitutively and inducibly expressed in plant organs. Antimicrobial peptides (AMPs) from plants are a large group of molecules with a wide or narrow-specific antimicrobial activity (antifungal, antibacterial), with various mechanisms of action. Most plant AMPs are cysteine-containing and disulfide-forming peptides, but some of the peptides are linear, typically folding into a dimensional helical configuration that is close to peptide toxins derived from spiders, insects, mammalians, and some higher fungi [1,2,3]. Proline/hydroxyproline-containing peptides from plants are usually represented by molecules with a signal function, inducing a defense gene expression, upon pathogen inoculation or under the attack of pests. Initially, these polypeptides were isolated from Solanaceae plants (tobacco, tomato, potato, and petunia) and it was shown that the precursor was post-translationally-modified through the hydroxylation of proline residues, followed by a specific enzyme cleavage to create mature peptides [4]. These components induce biochemical reaction cascades that lead to plant cell wall lignification and the expression of a defense gene (e.g., preliminary exohydrolase inhibitors) [5,6,7]. Previously, we reported that two homologous proline/hydroxyproline glycopeptides were isolated from dandelion (*Taraxacum officinale* Wigg.) flowers. Complete primary structures were de novo estimated by a combination of Edman sequencing and tandem MS/MS fragmentation; they were structurally characterized by pentoses-linked hydroxyproline residues and folded as a polyproline helix, based on the circular dichroism (CD) spectra data [8]. The mode of the antifungal action, displayed by the peptides, was high-specific towards well-colorized plant pathogenic fungi that could consist of an inhibition of melanin biosynthesis pathway [9]. Plant hairpin-like peptides (α-hairpinins) are represented by a new family of defense peptides with antimicrobial [10,11,12,13,14], protease inhibition [15,16], and ribosome-inactivating properties [17], which can also bind to DNA [18]. As antimicrobials (antifungals), α-hairpinins cause a static inhibitory effect towards the plant pathogenic fungi that leads to a suppression of spore germination and mycelium growth. For the antimicrobial EcAMP1 α-hairpinin previously isolated from the wild cereal grass *E. crusgalli*, we first determined its three-dimensional structure by NMR, in solution, identifying thirty-seven amino acid residues, two disulfide bonds, and two α-helices with a β-hairpin site generating the hydrophobic core, which involves cysteines and five amino acid residues (Met-12, Pro-19, Val-22, Val-26 and Arg-8) [12]. In this work, we describe the novel native EcAMP1 homolog with a modification of the proline-19-hydroxyproline, and report its structure, molecular modeling, and biological activity.

## 2. Results

**Figure 1:**
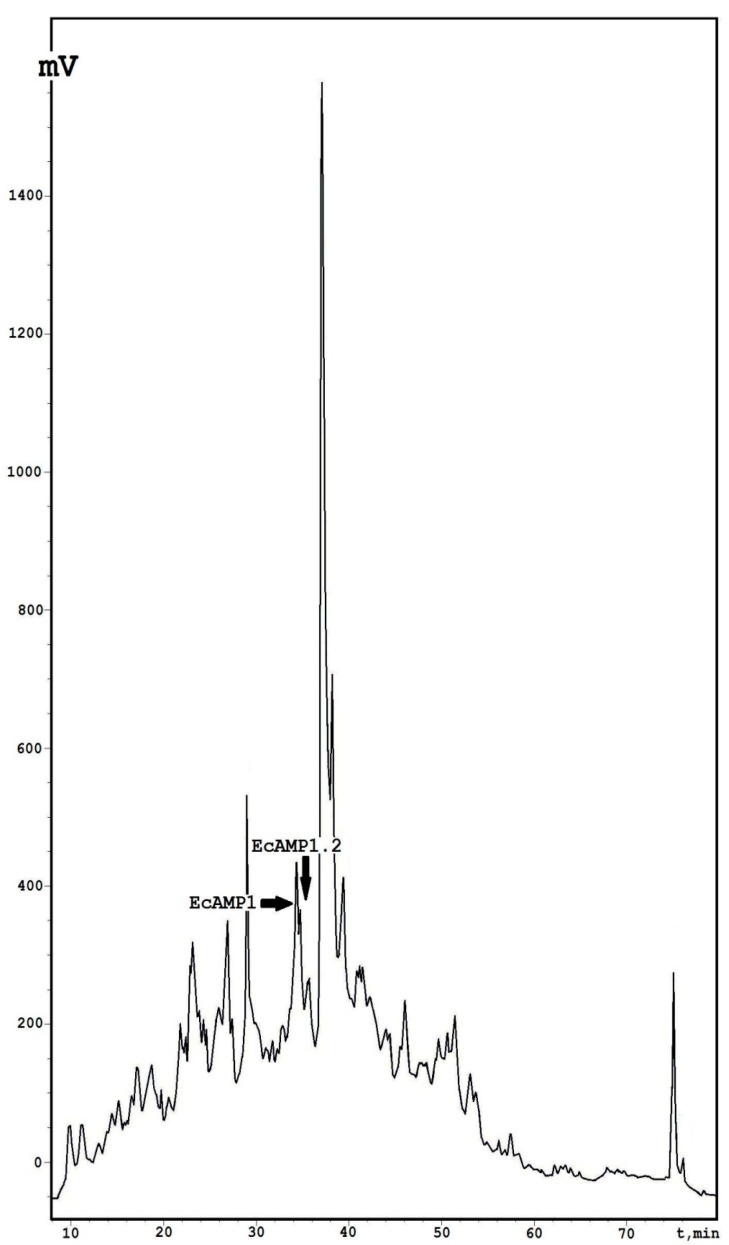
Isolation of the EcAMP1 peptides by analytical reversed-phase high performance liquid chromatography (RP-HPLC). Peaks corresponding to the EcAMP1 and EcAMP1-Hyp are indicated by the black arrows.

From the seeds of the barnyard grass (*E. crusgalli*), according to a previously developed algorithm [19] based on the two-step high-pressure liquid chromatography, a polypeptide profile was generated (Figure 1). This set included, approximately, ten main components between 10–75 min that were characterized by mass spectrometry, in our previous studies [12]. The compound eluted from the column at 35 min, was identified as EcAMP1 (*m*/*z* = 4275.2 Da) and α-hairpinin with antifungal activity.

**Figure 2:**
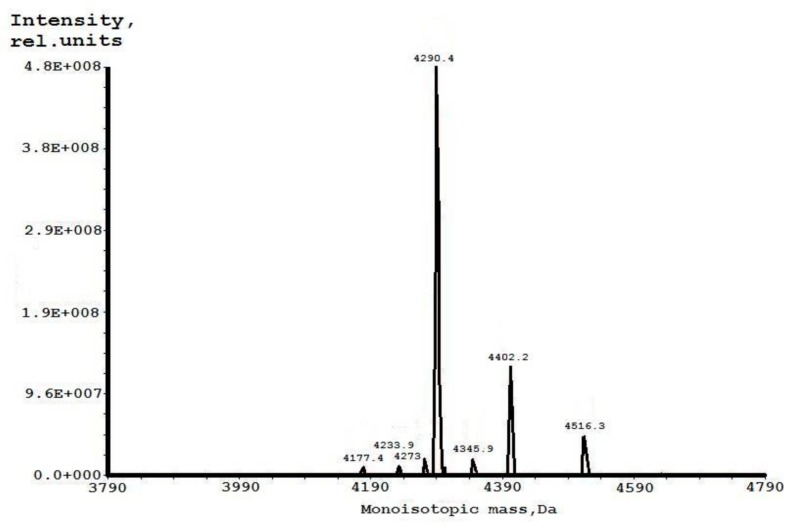
Analysis of the EcAMP1-Hyp.

**Figure 3:**
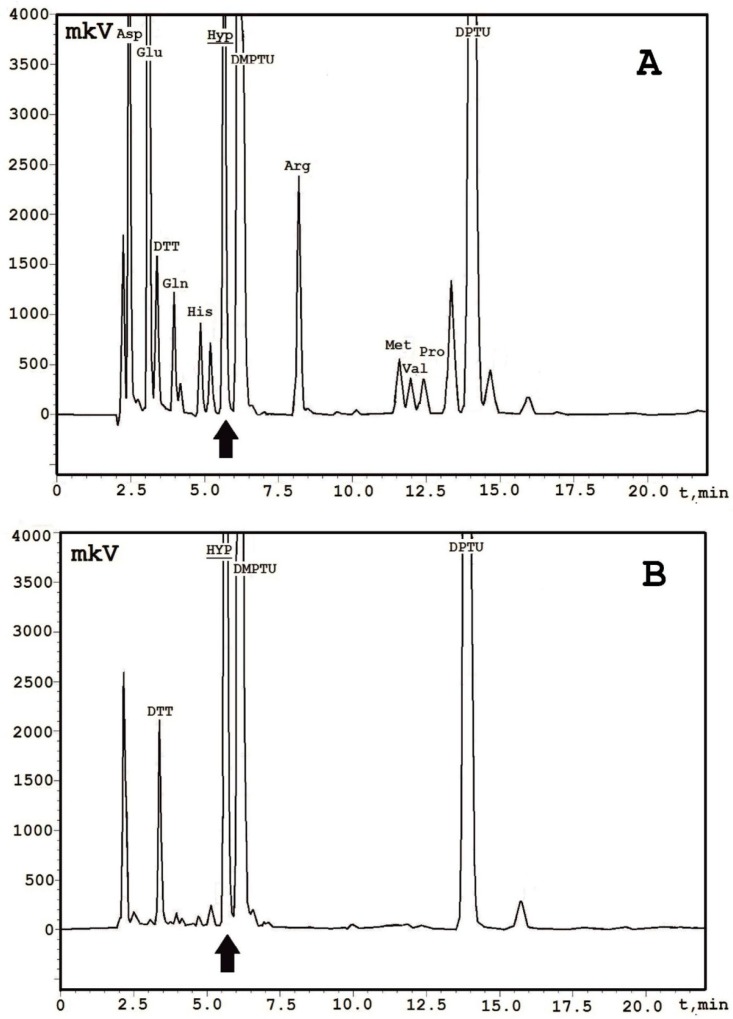
Identification of the hydroxyproline residue by the Edman automated sequencing. **A**—chromatographic separation of the PTH-amino acid derivatives generated on the 19th cycle of the EcAMP1-Hyp analysis, dithiothreitol (DTT); dimethylphenylthiourea (DMPTU); diphenylthiourea (DPTU). **B**—chromatographic separation of a PTH-amino acid derivative generated on the 1st cycle of an l-hydroxyproline analytical standard. A peak corresponding to the PTH-hydroxyproline is marked by the black arrow.

While this purification method was applied, one more *m*/*z* was determined as an admixture with a monoisotopic molecular mass of 4291.4 Da, which was greater than +16 Da (Figure 2). This peak was manually collected, re-chromatographed, and analyzed by the Edman sequencing. Its complete amino acid sequence was determined, and it contained 37 amino acid residues identical to EcAMP1, except having only a single amino acid substitution at position 19. The phenylthiohydantoin (PTH)-derivative of the amino acid residue located at 19 cycles of the Edman sequencing was not calibrated with any standard peak. Therefore, it was supposed that this component was non-protein encoded. We compared the typical chromatographic profiles for the most-widespread natural PTH-derivatives of the non-standard amino acids, based on the paper by authors of Reference [19], and discovered a concordance to hydroxyproline. To confirm this hypothesis, we sequenced an l-hydroxyproline analytical standard followed by the comparison of two the profiles received. As a result, we looked for exact compliance of the peaks, corresponding to PTH-hydroxyproline that were eluted from the analytical C_18_ reversed-phase (RP) HPLC, at 5.7 min (Figure 3). Based on the MS data, we concluded that the hydroxyproline residue was not glycosylated, as previously reported for the To-Hyp1/2 peptides from dandelion flowers [8] and some other plant hydroxyproline-rich polypeptides [20].

To understand if there is a difference in antifungal activity, we performed comparative flash “one-point” testing of the isolated EcAMP1 and EcAMP1-Hyp, and found that the hydroxyproline-modified peptide was significantly less active against the *Fusarium solani*, than the non-modified (at 4 µM concentrations mycelium growth was inhibited by 38% and 52%, respectively). To study the modified peptide in more detail, solid-phase synthesis of EcAMP1-Hyp was conducted, followed by the S-S-bonds locking and the RP-HPLC purification. In this method, 4-trans-hydroxyproline was used because this residue was the most widespread among proteins and peptides isolated from living organisms, in particular, the shellfish [21]. After lyophilization, 4.2 mg of the peptide, with oxidized SH-groups, was obtained. Recombinant EcAMP1 was received in heterologous expression in *Escherichia coli*, as mentioned earlier [22].

**Figure 4:**
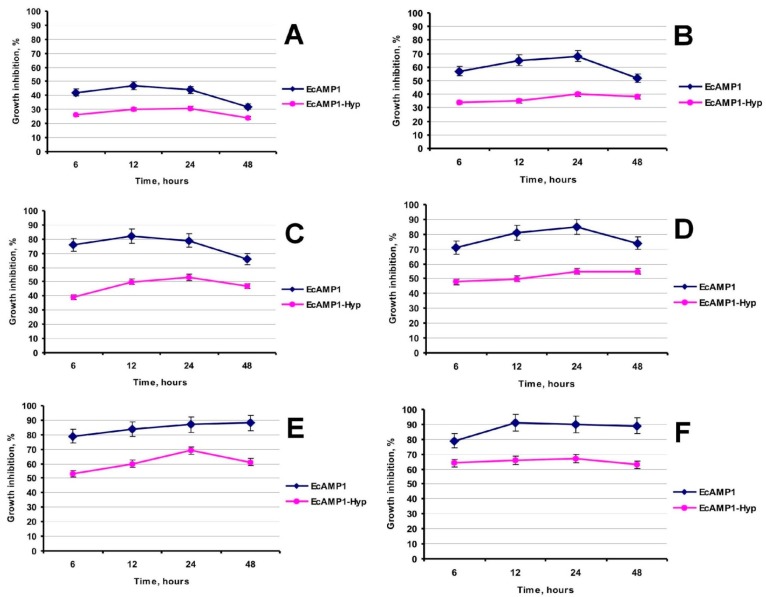
Comparative time-dependent fungistatic activity of the EcAMP1 and EcAMP1-Hyp: (**A**)—active peptide concentration of 2 µm; (**B**)—4 µm; (**C**)—8 µm; (**D**)—16 µm; (**E**)—32 µm and (**F**)—64 µm.

The time-dependent dynamics of *F. solani* mycelium growth inhibition in the presence of various concentrations of EcAMP1 or EcAMP1-Hyp was measured by optical density. We found that EcAMP1 was more active than the modified analog at all measured time and dose parameters. In more detail, at short time dynamics (6–12 h) both peptides demonstrated a more significant inhibitory effect at sub-inhibitory (IC_min_) and half-inhibitory (IC_50_) concentrations (2 and 4 µM, respectively, relative to the EcAMP1 assayed), and it was more quantitatively expressed at the increased concentrations (16–64 µM) (Figure 4, Table 1).

**Figure 5:**
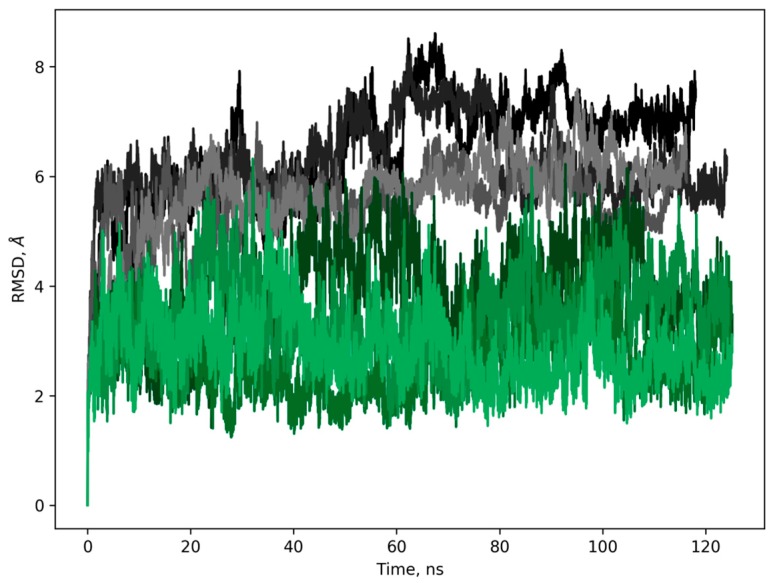
Root mean square deviations (RMSD) of the heavy atoms from the loop residues 15–19. EcAMP1 replicates are in gray and the EcAMP1-Hyp replicates are in green.

**Figure 6:**
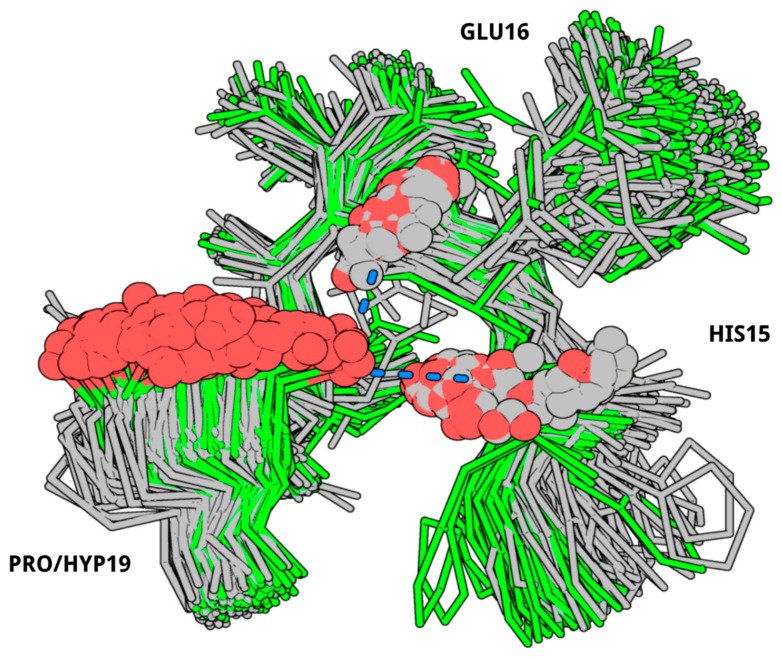
Clustered loop conformations. Spheres depict the donors and the acceptors of the hydrogen bond (WT is in grey, HYP is in green with red spheres, hydrogen bonds are colored with blue.

To determine which differences in the spatial structure of the peptide could lead to the significantly reduced antifungal activity, we undertook 3D modeling of the 4-trans-hydroxyproline orientation and the flexibility in the hairpin site of the molecule. As a result, during the simulation of the EcAMP1 (“wild-type”, WT), the loop deviation, from the starting position, was much larger (from 4 to 8 Å with an average about 6.5 Å) than during the simulation of the EcAMP1-Hyp (“hydroxyproline”, HYP) (from 2 to 6 Å with an average about 3.5 Å) (Figure 5). Closer analysis of the typical conformations, after cluster analysis, showed that the HYP loops were more tightly packed than in the WT. The reason of this packing was the formation of hydrogen bonds between the hydroxyl group of the HYP and the backbone oxygen atoms of the loop residues His-15 and Glu-16 (Figure 6).

**Figure 7:**
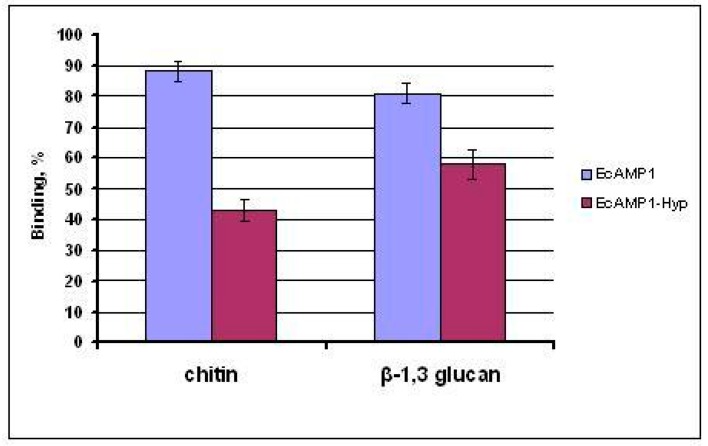
Quantitative in vitro binding of the EcAMP1 and the EcAMP1-Hyp with commercial carbohydrates.

Next, both peptides were checked for their binding with commercial polysaccharides, chitin, and β-1.3-glucan in vitro using the “batch-method”. As a result, the EcAMP1-Hyp had reduced binding with both carbohydrates, relative to the original AMP (Figure 7).

## 3. Discussion

In this work, we reported a novel high homologous hairpin-like peptide from the EcAMP family, isolated from the barnyard grass (*E. crusgalli*) seeds and having a natural single amino acid substitution from the proline to the hydroxyproline, at position 19 of the polypeptide chain. This modified amino acid residue was determined by the N-terminal Edman sequencing, followed by a comparison with a non-coded l-hydroxyproline analytical standard. The detected peptide possessed weaker antifungal activity towards the plant pathogenic fungus *F. solani*, one of the most EcAMP1-sensitive cultures [12,22]. It is interesting that only a single amino acid substitution (Pro19Hyp) was able to reduce the antifungal effect. This finding could be important for understanding the structure-function relationships and molecular interaction between the defense polypeptides and the target microbes. Previously, we determined a full-length gene structure encoding hairpin-like peptides from the *E. crusgalli* [23]. This was the first report to identify a hydroxyproline-containing derivative, among plant hairpin-like peptides. Previously, there were only two papers describing α-hairpinins from cereal crops, except for *E. crusgalli*—MBP-1 from maize (*Zea mays*) [10], and Tk-AMP-X1/X2 [13] from hexaploid wheat (*Triticum kiharae*), which were both cultivars. It is typical that barnyard grass, which is represented as a annual cereal, is a donor for the whole family of α-hairpinins, titled EcAMPs. These peptides were involved in two highly active fungistatic molecules, EcAMP1 [12,22] and EcAMP3 [23], and EcAMP2, which is not active, at all [24]. None of the peptides was found to have any posttranslational modifications, excepting disulfides. However, for EcAMP2, through acidic hydrolysis, five *C*-terminal residues were detected to have been lost [24]. This phenomenon could be explained by the cleavage of some non-specific carboxypeptidase, but this has not been reported for any other isolated peptides. It is interesting that the hydroxyproline-containing α-hairpinin was found only for the EcAMP1, but not for the other molecules, which were produced from a whole protein-precursor, after maturing [23], in manner similar to most hairpin-like defense peptides, isolated from monocots and dicots [11,13,14]. Compared to the EcAMP1, EcAMP1-Hyp displayed statistically weaker fungistatic activity towards a model strain of the *F. solani*. This was observed for the time and concentration parameters. However, it is important to note that, unlike the EcAMP1, the incubation of fungal mycelium with EcAMP1-Hyp, led to smoother time-dependent dynamics of inhibition, whereas, the maximum activity, after the incubation with EcAMP1, was composed of 12–24 h, and decreased by 48 h, as a rule. Our findings suggest a critical role for the Pro-19 residue, which is located in the β-hairpin site and is strictly involved in interaction with some molecular targets. Previously, we discovered the cellular mechanism of the EcAMP1 interaction with *F. solani* spores by confocal fluorescence, scanning two-photon high resolution (4Pi), and atomic force microscopy. In the short term (1–6 h), most of the peptide quantity accumulated onto the spore cover (the cell wall and plasma membrane, which could not be differentiated using low-resolution optical microscopy techniques, 130 nm maximum). Subsequently, some of the peptide was internalized into the cytosol [12,22]. We suggest that, at least, one mode of the peptide action was associated with fungal spore cover, mainly cell wall consisting of carbohydrates (chitin, β-1,3/1,6 glucans, generally), and therefore, we conducted in vitro binding of both peptides to commercially available chitin and β-1,3 glucans. As mentioned above, compared to the EcAMP1, EcAMP1-Hyp had reduced binding affinity with all of the tested polymers (43% vs. 58%). Thus, for chitin, this effect was significantly more expressed, that is, it could be supposed that chitin was the dominant structure polymer in the fungal cell wall architecture [25]. This finding confirmed that Pro-19 was also important for binding to carbohydrates located on the spore cell wall and it was chiefly realized in the fungistatic action representing the hyphal growth inhibition. Our observations were further confirmed by 3D modeling and molecular dynamics (MD) calculations. The loop region in the EcAMP1-Hyp structure had less flexibility, which specifically acted on the hydrophobic core mobility, and, the properly possible initial interactions, with some target polymers, were integrated to the cover [12]. Moreover, a hydroxyl group associated with the proline-19 residue might decrease the total charge of the whole molecule, as well as the loop region. This could also lead to a reduction of the peptide binding level to the cell wall, which preliminary consists of negative-charged polysaccharides [26]. The structure-function relationships between the AMPs and the target microbes (bacteria, fungi) are being studied intensively and have attracted the attention of scientists around the world [27]. Concerning plant α-hairpinins, some studies have addressed local amino acid mutagenesis, which usually leads to altered biological activity. For instance, residue substitutions in the hairpin region of the Tk-AMP-X2 antimicrobial peptide, from *T. kiharae* seeds, added a novel potassium channel blocker function [28], which is analogous to toxins from scorpion venom; mutagenesis of tryptophan and all cysteine residues that lead to disulfide unlocking and structural reconstruction in MBP-1, from maize kernels, resulted in a total decrease of antibacterial activity and DNA-binding [18]. For hairpin-like peptides from buckwheat (*Fagopyrum tataricum*), site-directed manipulations of the Arg-21 residue, which is critical for trypsin and trypsin-like inhibition in α-hairpinins [15,16], lead to a complete loss of this activity, but not the antifungal activity [29].

These data are important for further understanding of the plant’s innate immunity to biotic stress factors, based on defense peptides.

## 4. Materials and Methods

### 4.1. Biological Material

The seeds of *E. crusgalli* were collected in the Krasnodar region (Russian Federation). They were stored at a dried, ventilating room, at 15 °C. *Fusarium solani* TSKHA-4 strain was taken from the collection of the Plant Pathogenic Fungi from the Department of Plant Protection Timiryazev Russian State Agrarian University (Moscow, Russia).

### 4.2. Isolation of Hyp-Containing EcAMP1 from E. crusgalli Seeds

The peptide was isolated from the *E. crusgalli* seeds, according to the protocol published previously [24]. Acid-water extraction of the crushed seeds, followed by the precipitation of proteins and peptides, by cooled acetone, was carried out. After that, a two-step, high-pressure hydrophobic chromatographic purification, including a solid-phase extraction (SPE) and analytical reserved-phase HPLC (Phenomenex, Torrance, CA, USA) were conducted.

### 4.3. Mass Spectrometry

Molecular mass of the peptides was measured by a matrix-assisted laser desorption/ionization (MALDI) time-of-flight (TOF) mass spectrometry, on an Autospeed MALDI-TOF instrument (Bruker Daltonics, Bremen, Germany), in a positive ion mode. 2,5-Dihydroxybenzoic acid (Sigma, Ronkonkoma, NY, USA) was used as a matrix. Mass spectra were analyzed with FlexAnalysis software (Bruker Daltonics, Bremen, Germany).

### 4.4. N-Terminal Sequencing

The complete primary structure of the peptide was determined b the Edman degradation [30], on an automated PPSQ-33A protein sequencer (Shimadzu Corp., Kyoto, Japan), according to the manufacturer’s protocol. Phenylthiohydantoine (PTH)-derivatives of amino acids were estimated with LabSolutions software (Shimadzu Corp., Kyoto, Japan). Three hundred picomoles of the l-hydroxyproline analytical grade (Serva, Heidelberg, Germany), was used as the standard.

### 4.5. Peptide Synthesis

Solid-phase peptide synthesis was performed on automatic peptide synthesizer (Agilent technologies, Santa Clara, CA, USA) based on the Gilson automated liquid handler system (Gilson Scientific Ltd., Dunstable, UK), according to Gilson application note 228 (Gilson Scientific Ltd., Dunstable, UK). Preparative purification was carried out on a Gilson HPLC system (322 pump with GX-271 liquid handler, Gilson Scientific Ltd., Dunstable, UK) equipped with an YMC Triart 10 μm (150 × 30 mm) column and a UV detector at 210 and 280 nm. The peptides were eluted with a linear H_2_O-MeCN gradient (from 5% to 35% of MeCN), with 0.1% trifluoroacetic acid, at a flow rate of 30 mL/min. HPLC-MS analysis was performed using Thermo Finnigan LCQ Deca XP ion trap instrument (Waltham, MA, USA) with Thermo Accela UPLC system equipped with Phenomenex Jupiter C_4_ 5 μm (150 × 2.1 mm) column. Detection was achieved by UV-VIS diode array detector (DAD) (Thermo Fisher Scientific, Waltham, MA, USA) and a full scan mass spectrometry (electro spray ionization+, 150–2000 Da, Thermo Fisher Scientific, Waltham, MA, USA). A polystyrene-PEG 2000 block-copolymer resin (Sigma-Aldrich, St. Louis, MO, USA), modified with carboxy-trytil linker (Tentagel HL-TRT, Rapp Polymere, Tübingen, Germany), Fmoc-protected amino acids from Iris Biotech (Marktredwitz, Germany) were used, except for Fmoc-Hyp(tBu)-OH, which was from Novabiochem (Sigma-Aldrich, St. Louis, MO, USA). Acetyl chloride, 4-methyl piperidine, diisopropylethylamine, sym-collidine and HATU were from Acros Organics (Morris, NJ, USA) and Sigma-Aldrich (St. Louis, CA, USA), respectively. All reagents and solvent were used without additional purification. *C*-terminal amino acid was attached to the Ac-Cl activated resin, in the presence of Huenig’s base during 2 h. Peptide assembly was performed by Fmoc-methodology, using HATU/collidine activation. An 8-fold excess of amino acids was used within 30 min of the condensation time. After the synthesis, the protected peptidyl-polymer was washed with diethyl ether, then dried, and treated with trifluoroacetic acid/dithiothreitol/deionized water/triispropylsilane (TFA/DTT/H_2_O/TIS) 150/4/3/0.5 (weight proportion) mixture. Fifteen milliliter of the mixture was applied to 1 g of peptidyl-polymer, during 2 h. Then the solution was filtered out, the dry peptide was precipitated with a ten-fold volume of diethyl ether and kept at 4 °C, for 8 h. The precipitated peptide was centrifuged, washed three times with diethyl ether, and then dried under vacuum. Crude peptide was purified by HPLC, and then lyophilized. Pure linear peptide was dissolved in 50 mM of ammonium bicarbonate in water/acetonitrile 90:10, to a final concentration of 0.5 mg/mL. The resulting solution was stirred in air, overnight, then acetonitrile was evaporated under vacuum, and the residual solution was acidified by 1% *v*/*v* acetic acid and injected for RP-HPLC. After purification, the desired fractions were lyophilized and analyzed by ultra performance liquid chromatography-mass spectrometry (UPLC-MS) (Agilent technologies, Santa Clara, CA, USA). The obtained molecular mass of the peptide was very close to the theoretical calculations.

### 4.6. Obtainment of the Recombinant EcAMP1 in the E. coli System

This procedure was carried out according to the protocol designed, previously, for hairpin-like peptides from the *E. crusgalli* seeds [22,23].

### 4.7. Chitin- and β-1.3-Glucan-Binding Assay In Vitro

For these experiments, shrimp shell chitin (Sigma, Ronkonkoma, NY, USA) and β-1.3-glucan from barley (Sigma, Ronkonkoma, NY, USA) were used. To determine the quantitative binding of the EcAMP1 peptides to the carbohydrates, “batch”-method was applied, according to that previously described, for hevein-like AMP SmAMP3 [31].

### 4.8. Molecular Modeling

Initial structures were prepared, based on that of PDB ID 2L2R (available online: https://www.rcsb.org/structure/2L2R). HYP was manually modified with PyMol (DeLano Scientific, Palo Alto, CA, USA). Classical geometry optimization was performed for each system. Explicit solvent simulations in the amber99sb-ildn force field [32] were performed at Т = 300 K, under the control of a velocity rescaling thermostat [33]. These were done with isotropic constant-pressure boundary conditions, under the control of the Berendsen algorithm of pressure coupling [34], using an application of the particle mesh Ewald method for long-range electrostatics interactions (PME) [35]. A triclinic box of the TIP4P [36] water molecules was added around the peptide to a depth 15 Å, on each side of the solute. Negative charges of the systems were neutralized by addition of sodium cations, resulting in 0.15 M concentration of the ions. The system was divided into two temperature coupling groups—protein and water with ions. Eight independent replicas (4 for EcAMP1 and 4 for EcAMP1-Hyp) were created. Analysis was carried out by tools from the GROMACS 2016 software package (available online: www.gromacs.com) [37] and with Python scripts. Cluster analysis was performed with the adapted Affinity Propagation algorithm (available online: https://www.psi.toronto.edu/affinitypropagation/webapp/) [38].

### 4.9. Antifungal Activity

Antifungal activity of the peptides was tested against *F. solani* by microtiter plate assay, as described earlier [39]. Comparative time-dependent fungal growth dynamics, based on the measurements of the optical density (6, 12, 24 and 48 h), was provided at the active peptide concentrations received by the two-fold dilution way (2–64 µM), as mentioned previously [22]. Two main parameters (IC_50_ and IC_min_) were determined.

## Figures and Tables

**Table 1:** Antifungal activity of the EcAMP1-Hyp against the *F. solani*, compared with the native peptide EcAMP1, µM.

Peptide	IC_min_	IC_50_
EcAMP1-Hyp	2.9	5.4
EcAMP1	2.2	3.8
